# Comparison of the effect of skin closure materials on skin closure during cesarean delivery

**DOI:** 10.1371/journal.pone.0270337

**Published:** 2022-06-30

**Authors:** Ye Huang, Xinbo Yin, Junni Wei, Suhong Li

**Affiliations:** 1 School of Public Health, Shanxi Medical University, Taiyuan, Shanxi, China; 2 Department of Pathology, Children Hospital and Women Health Center of Shanxi, Taiyuan, Shanxi, China; 3 Xiangya Hospital, Central South University, Changsha, Hunan, China; Universiteit Hasselt Faculteit Wetenschappen, BELGIUM

## Abstract

**Objective:**

To compare the effect of skin closure materials on skin closure during cesarean delivery.

**Methods:**

We searched EMBASE、PubMed、Scopus、Cochrane CENTRAL for randomized controlled trials (RCTs) on the use of closure materials for skin closing effect during cesarean delivery. The outcomes were time to skin closure of dermal and epidermal layer, skin separation rate and wound complications(wound infection, hematoma,seroma, reclosure, readmission) reported as an odds ratio (OR) and surface under the cumulative ranking curve analysis (SUCRA) score.

**Results:**

Twenty -six RCTs met the inclusion criteria. In the network meta-analysis (NMA) for time to skin closure of dermal and epidermal layer, pooled network OR values indicated that staple (network SMD, -337.50; 95% CrI: -416.99 to -263.18) was superior to absorbable suture. In the Skin separation NMA, pooled network OR values indicated that the absorbable suture (network OR, 0.37; 95% CrI: 0.19 to 0.70) were superior to staple. In the wound complications NMA, pooled network OR values indicated that the no interventions were superior to staple.

**Conclusion:**

In conclusion, our network meta-analysis showed that the risk of skin separation with absorbable suture after cesarean delivery was reduced compared with staple, and does not increase the risk of wound complications, but the wound closure time would slightly prolonged.

## Introduction

Cesarean section has many indications, including emergency surgery to save the lives of mothers and infants in dystocia or other emergencies, as well as maternal desire [1]. The World Health Organization recommends that the cesarean section rate should not be higher than 15%, but the cesarean section rate in many countries is higher than this standard [2,3]. What sutures or suture combinations are used in any particular surgical case varies widely among surgeons [4]. The selection of skin closure materials is usually based on surgeon’s preference, institutional agreement, availability and cost of specific materials, or current interest in exploring a new technology based on technological progress [5]. Therefore, it is necessary to evaluate skin closure materials.

According to the different degradation modes of suture, it can be divided into absorbable suture and non-absorbable suture. Barbed suture is a single filament suture with thorns, without the need for surgical knots. Staple is a disposable skin stapler with the characteristics of high speed. Glue closes the skin with a liquid monomer that forms a firm tissue bond with the protective barrier [6]. There is controversy about the way of skin closure after cesarean delivery. Previous meta-analysis shows that absorbable suture reduces the risk of skin separation compared with suture staple, but increases the time of wound suture [7–9]. Compared with suture, the barbed suture reduces the time to skin closure of dermal and epidermal layer and total operative time without increasing blood loss or maternal incidence rate [5]. Therefore, the purpose of this study is to update the evidence through network meta-analysis (NMA) and compare the time to skin closure, incidence of skin separation and wound complications of different skin closure materials during skin incision suture in cesarean delivery.

## Methods

### Protocol

This NMA followed the guidelines outlined in the Preferred Reporting Items for Systematic Review and Meta-Analyses (PRISMA) report. The protocol used in this study was registered in the International Prospective Register of Systematic Reviews (Registration number: CRD42021249871, date: 2021-05-24).

### Search strategy

Two authors (Y.H. and XB.Y.) independently searched the Cochrane Central Database, PubMed, and EMBASE databases for randomized controlled trials (RCTs) on different kinds of skin closure materials for women after cesarean section skin closure from January1, 1997 to June1, 2021. A third author (SH.L.) was consulted to resolve differences through discussion, as appropriate.

The following is the PubMed search strategy:

(((((((((((((((((((((((((closur*) OR (sutur*)) OR (sutures[MeSH Terms])) OR (stapling or staples)) OR (surgical staplers[MeSH Terms])) OR (polydioxanone)) OR (polydioxanone[MeSH Terms])) OR (pds)) OR (polypropylene*)) OR (Polypropylenes[MeSH Terms])) OR (prolene*)) OR (polyglactin 910[MeSH Terms])) OR (polyglactin 910)) OR (ethilon)) OR (Nylons[MeSH Terms])) OR (catgut)) OR (catgut[MeSH Terms])) OR (steel)) OR (steel[MeSH Terms])) OR (vicryl)) OR (polyglycolic acid)) OR (polyglycolic acid[MeSH Terms])) OR (maxon)) OR (mersilene*)) OR (Barbed*)) AND ((((((((caesarean[Title/Abstract] OR cesarean[Title/Abstract]) AND (section[Title/Abstract] OR birth?[Title/Abstract] OR deliver*[Title/Abstract] OR surgery[Title/Abstract])) OR (((c‐section[Title/Abstract])) OR (childbirth[MeSH Terms])))AND (birth[Title/Abstract] OR childbirth[Title/Abstract]))) OR ((operative[Title/Abstract] OR surgical[Title/Abstract]) AND (birth*[Title/Abstract] OR deliver*[Title/Abstract]))) OR (("unnecessary cesarean*"[Title/Abstract] OR "unnecessary caesarean*"[Title/Abstract]))) OR (cesarean section[MeSH Terms])) OR (abdominal delivery[Title/Abstract]))

### Inclusion criteria

Randomized controlled trial involving women undergoing cesarean delivery.

#### Outcome

Time to skin closure of dermal and epidermal layer (seconds); Skin separation; Wound complications.

### Exclusion criteria

Nonrandomized or pseudo-randomized controlled trials; Incomplete or repeated data; Case studies; Reviews.

### Study selection

According to the inclusion and exclusion criteria, two authors (Y.H. and XB.Y.) independently identified potential studies among the studies yielded by the search strategy. A third author (SH.L.) was consulted to resolve differences through discussion, as appropriate.

### Data extraction

Two authors (Y.H. and XB.Y.) independently extracted relevant data using review manager software (version 5.3). In case of disagreements, the original text was re-checked again and discussed to come to an agreement. If no agreement was reached, the third author (SH.L.) was consulted for arbitration. We extracted the following data parameters: the name of the first author, number of patients, number of participants in each group, types of skin closure materials used, and type of the results (Time to skin closure of dermal and epidermal layer, skin separation and wound complications); moreover, the results were obtained for each arm.

### Risk and bias

Two authors (Y.H. and XB.Y.) independently assessed the risk and bias for each study using review manager software (version 5.3). The Cochrane Collaboration tool was used to evaluate the study quality based on the following six factors: sequence generation, allocation consideration, blind method, incomplete data, non-selective reporting of results, and other sources. Disagreements were resolved through arbitration with the third author (SH.L.).

### Outcomes

The primary outcome was time to skin closure of dermal and epidermal layer (seconds), defined as the skin closure of dermal and epidermal layer among women undergoing cesarean delivery, which was analyzed as a continuous outcome, and reported using the network standardized mean difference (SMD) and related 95% confidence interval (CrI). A negative network SMD value denoted a shorter suture time.

The secondary outcome was skin separation rate, defined as number of after skin closure materials are removed and need for reclosure cases. Therefore, treatment was analyzed as a binary outcome (successful or failed intervention) and reported using the network odds ratio (OR) and related 95% confidence interval (CrI). Consequently, treatment success was defined as a network OR (including the relevant 95% CrI) of 1.0 (unified).

The third outcome was wound complications, defined as the number of wound infection, hematomata, seroma, reclosure, readmission for wound complication causes after cesarean delivery. Therefore, treatment was analyzed as a binary outcome (successful or failed intervention) and reported using the network odds ratio (OR) and related 95% confidence interval (CrI). Consequently, treatment success was defined as a network OR (including the relevant 95% CrI) of 1.0 (unified).

### Statistical analyses

First, stataSE15 (64 bit) was used to draw a network diagram; subsequently, the relationship between the different skin closure materials was determined. Next, the heterogeneity analysis was conducted using the R software (version 3.6.1). According to the Cochrane handbook, when analyzing the data using a fixed-effect model, no heterogeneity was indicated for *P*-value >0.10, and an I^2^ value of 0%–40%. Heterogeneity was indicated by *P*-value <0.10, and I^2^ >75%, with data analysis using a random-effect model [10]. However, in this NMA, regardless of heterogeneity, we used a random-effect model to analyze the data reliability. Finally, NMA was conducted using the ADDIS software (version 1.16.8), which is based on a Bayesian hierarchical model. Node-splitting analysis was used to determine the model consistency. If the *P*-value is >0.05, the consistency model is used; otherwise, the inconsistency model is used [11]. Subsequently, the potential scale reduction factor (PSRF) analysis method was used to determine the model convergence. When the PSRF value is 1, the model is indicated as having approximate convergence, using the network OR and 95% CrI as the effect value [12].

## Results

### Study selection

**Fig 1:**
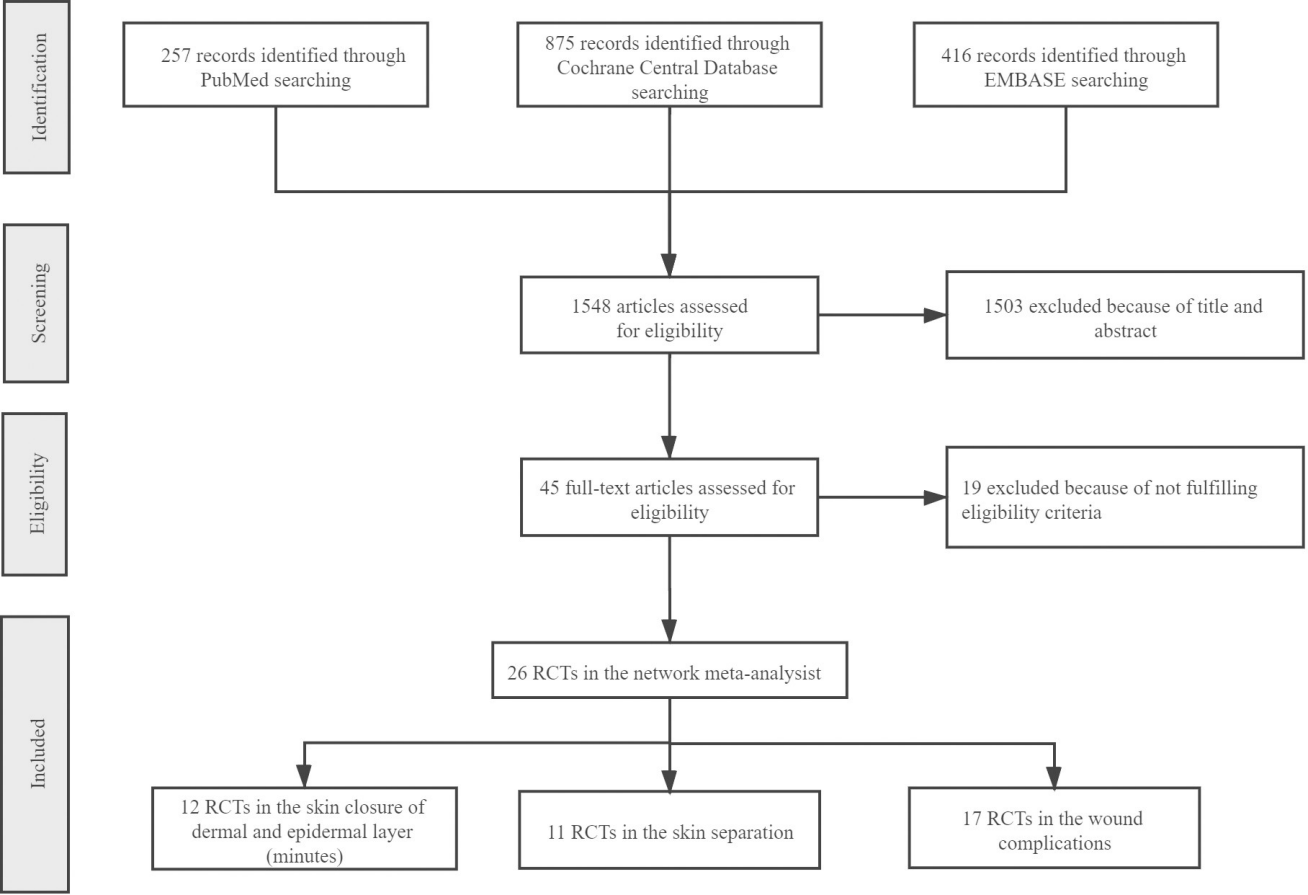
PRISMA process.

According to the PRISMA standard, 1,548 RCTs were retrieved from three databases based on a search strategy; of these, 45 eligible studies were screened after reviewing the abstracts. According to the inclusion and exclusion criteria, 26 RCTs were included (Fig 1).

### Characteristics of the included studies

This NMA included 26 RCTs, containing 23 two-arm studies, 3 three-arm studies. The studies were published between 1997 and 2020, with most of them published after 2010 (Table 1). The included studies reported eight antibiotic classes and doses, as well as placebo; Regarding the main outcome indicators, 12, 11, and 17 articles reported skin closure of dermal and epidermal layer (seconds), skin separation, and wound complications, respectively. We included 8,539 pregnant women who underwent cesarean delivery. The minimum and maximum sample sizes were 52 and 1,100 cases, respectively.

**Table 1:** Characteristics of the included studies.

Author, year	Country	Study size	Mean age in years (± SD)	Study design	Method of wound closure (n)	Suture material used	Incision type
**Frishman 1997 [25]**	USA	52	N/A	RCT	Absorbable sutures: 26 Staple: 26	N/A	Pfannenstiel incision
**Murtha 2006 [26]**	USA	188	Absorbable sutures: 27.9 (6.0) Barbed suture: 29.3 (6.2)	RCT; two-centre; single-blind	Absorbable sutures: 61 Barbed suture: 127	Absorbable sutures: 3–0 Polydioxanone Suture-II Barbed suture: Quill™ Medical bidirectional patternbarbed suture	Pfannenstiel incision
**Gaertner 2008 [27]**	Switzerland	1100	Absorbable sutures: Group A 31.1 Group B 30.3 Staple: Group C 32.5 Group D 31.6	RCT; single-centre; non-blinded	Absorbable sutures: 49 Staple: 51	Vicryl 3–0	Pfannenstiel incision
**Rousseau 2009 [13]**	Canada	101	Absorbable sutures: 30.7 (5.4) Staple: 30.6 (3.9)	RCT; single-centre; single blind	Absorbable sutures: 52 Staple: 49	3–0 polyglactin	Pfannenstiel incision
**Basha 2010 [28]**	USA	416	Absorbable sutures: 29.0 (5.7) Staple: 28.9 (6.1)	RCT; single-centre; non-blinded	Absorbable sutures: 219 Staple: 197	4–0 poligle-caprone	Pfannenstiel, vertical incision
**Cromi 2010 [29]**	Italy	158	Absorbable sutures: Group A: 33.3(5.4) Group B: 33.4(4.5) Group C: 34.1(4.5) Staple: 32.5 (4.8)	RCT; single-centre; single-blind	Absorbable sutures: 118 Staple: 40	3–0 glyconate or polyglycolic acid	Pfannenstiel incision
**Rengerink 2011 [30]**	N/A	133	N/A	RCT	Absorbable sutures: 67 Staple: 68	3–0 subcuticular poliglecaprone (Monocryl)	N/A
**Chunder 2012 [36]**	South Africa	1100	Absorbable sutures: median: 25 (range 19–31) Staple: median: 26 (range 18–29) Nonabsorbable sutures: median:24 (Range 18–32)	RCT; single-centre; non-blinded	Absorbable sutures: 361 Staple: 373 Nonabsorbable sutures: 366	Absorbable sutures: Polyglycolic acid Nonabsorbable sutures: nylon	Pfannenstiel incision
**De Graaf 2012 [14]**	Netherlands	124	Absorbable sutures: Group A 33.3(3.5) Group B 31.6 (4.7) Staple: Group C 31.4(4.1) Group D 31.3(4.9)	RCT; two-centre; single-blind	Absorbable sutures: 64 Staple: 60	3–0 polyglactin	Pfannenstiel incision
**Figueroa 2013 [31]**	USA	350	Absorbable sutures: 26.9 (5.9) Staple: 26.7 (6.1)	RCT; single-centre; non-blinded	Absorbable sutures: 171 Staple: 179	4–0 poliglecaprone	Pfannenstiel, vertical incision
**Huppelschoten 2013 [15]**	Netherlands	145	Absorbable sutures: Median 32 (range 21–42) Staple: Median: 31 (range 21–45)	RCT; single-centre; single blind	Absorbable sutures: 68 Staple: 77	3–0 poligle-caprone	Pfannenstiel incision
**Abdus-Salam 2014 [16]**	Nigeria	106	Absorbable sutures: 31.1 (4.27) Staple: 31.6 (4.5)	RCT; single-centre; single-blind	Absorbable sutures: 53 Staple: 53	2–0 polyglycolic acid	Pfannenstiel incision
**Mackeen 2014 [17]**	USA	746	Absorbable sutures Median: 31.0(IQRb 26.9– 35.4) Staple: Median: 31.0 (IQRb 26.4– 35.6)	RCT; multi-centre; single blind	Absorbable sutures: 370 Staple: 376	4–0 poligle- caprone/polyg- lactin	Low transverse incision
**Vats 2014 [32]**	India	90	N/A	RCT; single-centre; non-blinded	Absorbable sutures: 60 Nonabsorbable sutures: 30	Absorbable sutures: poliglecaprone 25/ polyglactin 910 Nonabsorbable sutures: polyamide	N/A
**Hasdemir 2015 [18]**	Turkey	250	Absorbable sutures: 27.8(5.2) Nonabsorbable sutures: 27.9(5.3)	RCT; single-centre; non-blinded	Absorbable sutures: 108 Nonabsorbable sutures: 142	Absorbable sutures: 3.0 Vicryl Rapide [polyglactin 910 Nonabsorbable sutures: 3.0 Prolen	Pfannenstiel incision
**Dhama 2016 [19]**	India	156	N/A	RCT; single-centre; non-blinded	Absorbable sutures: 50 Nonabsorbable sutures: 54 Staple: 52	Absorbable sutures: vicryl No Nonabsorbable sutures: nylon	N/A
**Fitzwater 2016 [37]**	USA	350	Absorbable sutures: 26.8(5.9) Staple: 26.7(6.1)	RCT; single-centre; single blind	Absorbable sutures: 171 Staple: 179	4–0 Monocryl	Pfannenstiel incision
**Daykan 2017 [6]**	Israel	104	Absorbable sutures: 34.44±4.9 Glue: 35±4.3	RCT; single-centre; non-blinded	Absorbable sutures: 52 Glue: 52	Absorbable sutures: Glue	N/A
**Grin 2018 [20]**	Israel	70	Absorbable sutures: 32.9 (6.1) Barbed suture: 32.4 (5.4)(6.2)	RCT; single-centre; single blind	Absorbable sutures: 35 Barbed suture: 35	Absorbable sutures: Polyglactin absorbable suture (Vicryl™, Ethicon) Barbed suture: Tensile strength size 1–0 absorbable Barbed suture (Stratafix™ Spiral PDO, Ethicon)	N/A
**Peleg 2018 [21]**	Israel	102	Absorbable sutures: 33(5.0) Barbed suture: 32.2(6.2)	RCT; single-centre; non-blinded	Absorbable sutures: 51 Barbed suture: 51	Absorbable sutures: Conventional coated size 1Polyglactin 910 braided sutures (Vicryl Plus™, Ethicon) Barbed suture: PDO monofilament Barbed suture size 2 (Stratafix™ Spiral PDO,Ethicon)	Pfannenstiel incision
**Zaki 2018 [34]**	USA	238	Absorbable sutures: 31.4 (5.3) Staple: 31.3 (5.6)	RCTb; multi-centre; non-blinded	Absorbable sutures119 Staple: 119	4–0 polyglactin; 3–0 poligle- caprone	Pfannenstiel, vertical incision
**Madsen 2019 [22]**	USA	206	Absorbable sutures: Median: 30 (IQRb 27–33) Staple: Median: 31 (IQRb 27–34)	RCT; single-centre; non-blinded	Absorbable sutures: 103 Staple: 103	3–0 poligle- caprone	Low transverse incision
**Zayed 2019 [23]**	Egypt	100	N/A	RCT; single-centre; single blind	Absorbable sutures: 50 Barbed suture: 50	Absorbable sutures: Polyglactin 910 (Vicryl™, Ethicon) Barbed suture: No 1, 36 × 36 cm, PDO double-armed suture (Stratafix™ SpiralPDO Ethicon)	Pfannenstiel incision
**Poprzeczny 2020 [38]**	South Australia	849	Absorbable sutures: 31.56 (5.32) Nonabsorbable sutures: 31.26 (5.73)	RCT; single-centre; single blind	Absorbable sutures 422 Nonabsorbable sutures: 427	Absorbable sutures: Caprosyn™ Nonabsorbable sutures: Prolene™	N/A
**Nayak 2020 [24]**	India	300	Absorbable sutures: 26.5(3.8) Staple: 27.0(4.3) Nonabsorbable sutures: 26.5(4.1)	RCT; single-centre; non-blinded	Absorbable sutures: 102 Staple: 100 Nonabsorbable sutures: 98	Absorbable sutures: 3–0 poligle-caprone; 2–0polyamide Nonabsorbable sutures: nylon	Low transverse incision
**Rodel 2020 [35]**	USA	180	Absorbable sutures: 28.0 (25.3–34.0) Staple: 28.0 (26.5–29.5)	RCT; two-centre; single-blind	Absorbable sutures: 90 Staple: 90	Monofilament (Monocryl) Braided absorbable (Vicryl)	Pfannenstiel, vertical incision

### Risk-of-bias and quality-of-evidence assessments

**Fig 2:**
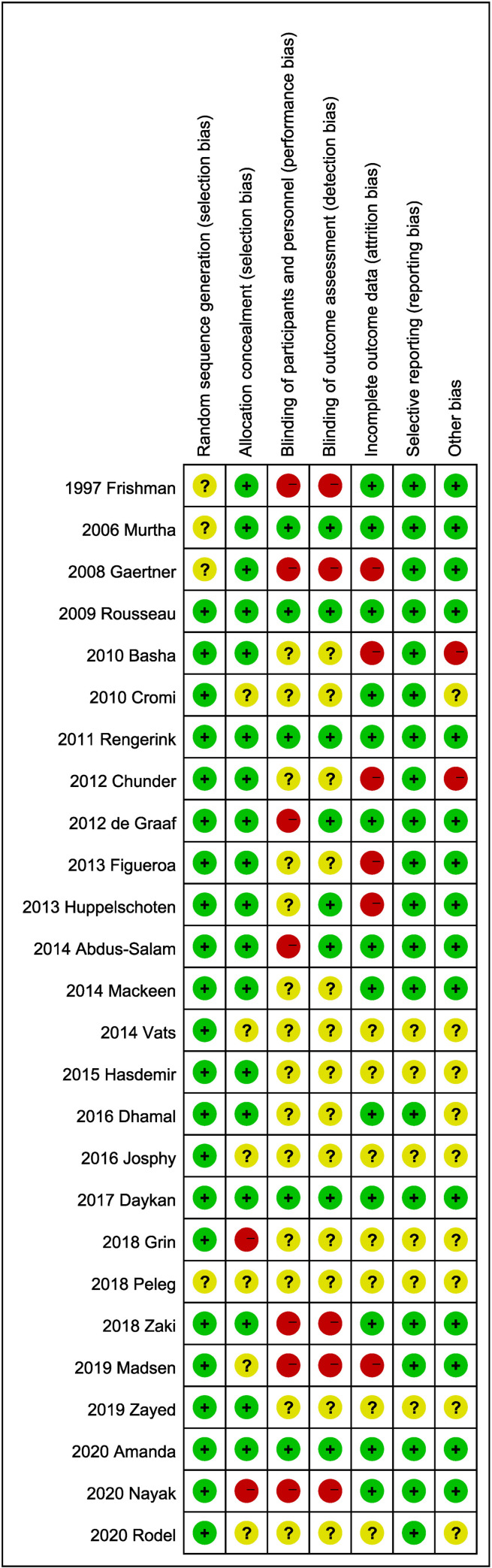
Risk-of-bias summary.

The risk-of-bias and quality-of-evidence assessments for the included study were performed using the Cochrane bias risk assessment tool. All the included trials were RCTs. Furthermore, 55% of the studies were rated as low risk of bias; moreover, 19 of the included RCTs described specific methods for generating a random sequence. Fig 2 shows the risk-of-bias summary of the included trials.

#### NMA for time to skin closure of dermal and epidermal layer (seconds)

**Fig 3:**
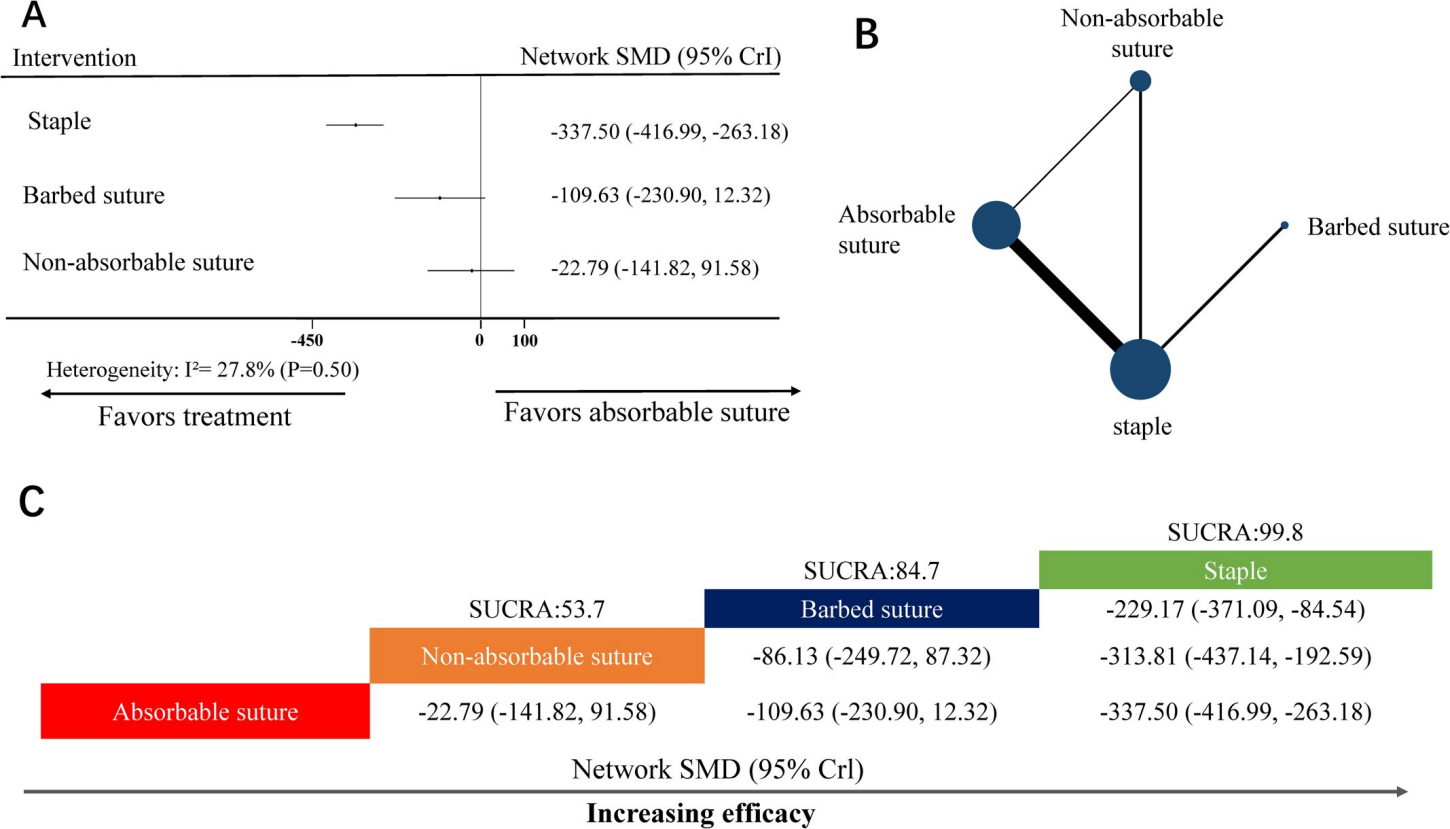
NMA for time to skin closure of dermal and epidermal layer (seconds). (A) Forest plot of the network meta-analysis comparing each intervention against absorbable suture. (B) Each node (blue circles) represents a unique skin closure material; moreover, the size of each node represents the included pregnant woman for the intervention. The connecting line indicates direct comparisons between both nodes. The width of each line represents the number of direct comparisons between interventions. (C) Schematic detailing the most efficacious skin closure material in NMA for time to skin closure of dermal and epidermal layer (seconds), and the surface under the cumulative ranking curve analysis (SUCRA) score. NMA, network meta-analysis; SMD, network standardized mean difference; CrI, confidence interval.

The NMA for time to skin closure of dermal and epidermal layer (seconds) included 12 RCTs [13–24] (10 two-arm studies, 2 three-arm studies) covering four skin closure materials (Fig 3A). Eight nodes were included in the NMA. Each node represented a unique skin closure material; further, the size of each node represented the included patients for the intervention (Fig 3B). Absorbable suture (11 head-to-head comparisons) and staple (10 head-to-head comparisons) were the most investigated skin closure material.

Heterogeneity analysis indicated no heterogeneity (I^2^-value = 27.8%, *P*-value = 0.5) (Fig 3A). Therefore, we used the random effect model to analyze the data.

In the NMA, the node-splitting analysis showed that *P*-values were >0.05 (S1 Table); therefore, we used the consistency-type model for data analysis. After 50,000 simulation iterations, the PSRF value was 1, which indicated that approximate convergence was achieved. Pooled network OR values indicated that staple (network SMD, -337.50; 95% CrI: -416.99 to -263.18) was superior to absorbable suture (Fig 3C). The SUCRA score revealed that the top-ranked classes for time to skin closure of dermal and epidermal layer (seconds) was staple (SUCRA score: 99.8; Fig 3C).

#### NMA for skin separation

**Fig 4:**
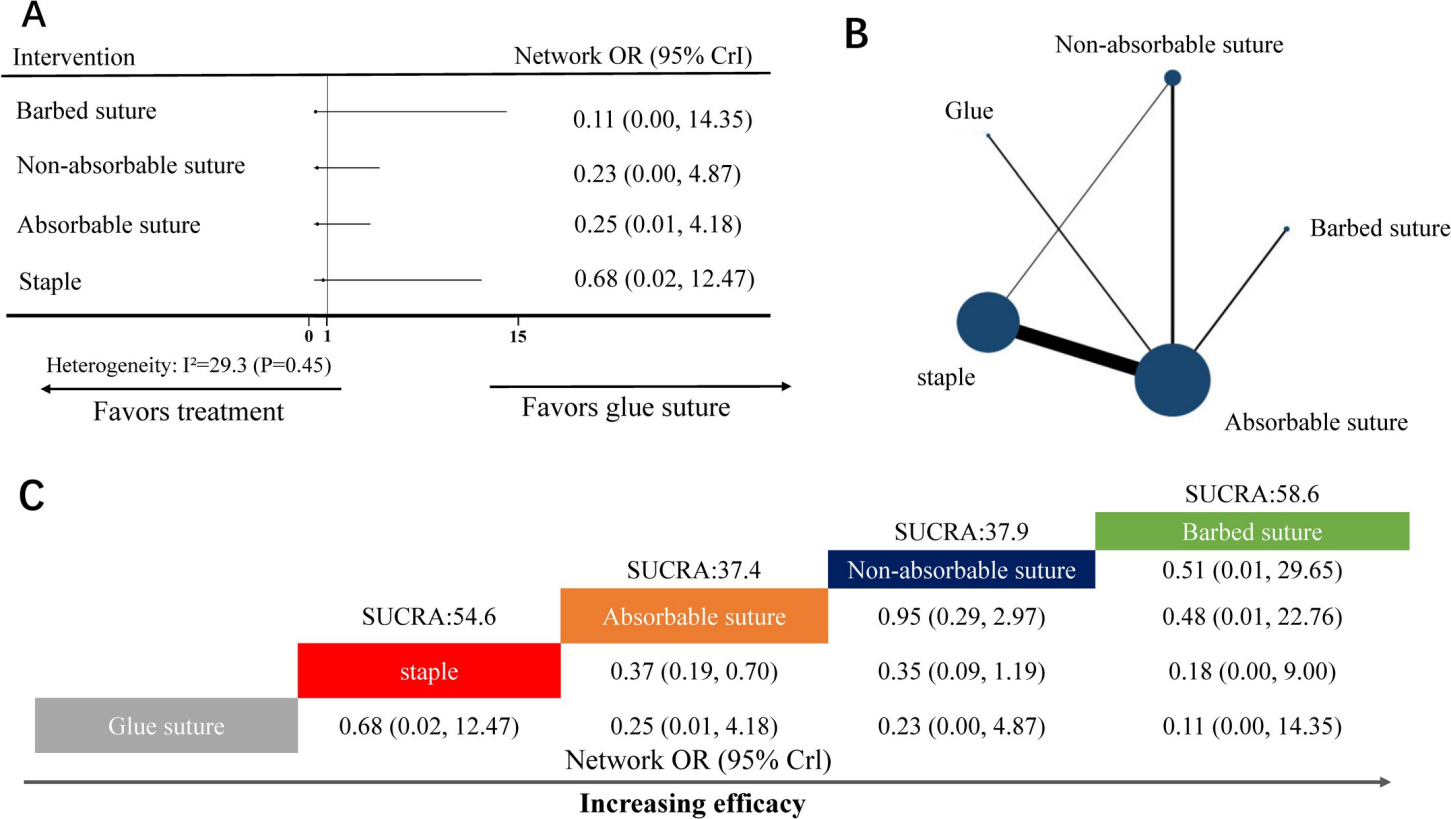
NMA for skin separation. (A) Forest plot of the network meta-analysis comparing each intervention against glue suture. (B) Each node (blue circles) represents a unique skin closure material; further, the size of each node represents the included pregnant woman for the intervention. The connecting line indicates direct comparisons between both nodes. The width of each line represents the number of direct comparisons between interventions. (C) Schematic detailing the most efficacious skin closure materials in NMA for skin separation, and surface under the cumulative ranking curve analysis (SUCRA) score. NMA, network meta-analysis; OR, odds ratio; CrI, confidence interval.

The NMA for Skin separation included 11 RCTs [17,18,22,24–35] (11 two-arm studies) covering five skin closure materials (Fig 4A). Nine nodes were included in the NMA. Each node represented a unique skin closure material; additionally, the size of each node represented the included patients for the intervention (Fig 4B). Absorbable suture (16 head-to-head comparisons) and staple (12 head-to-head comparisons) were the most investigated skin closure material.

Heterogeneity analysis indicated no heterogeneity (I^2^-value = 29.3%, *P*-value = 0.45) (Fig 4A). Therefore, we used a random effect model to analyze the data.

In the NMA, the node-splitting analysis showed that both *P*-values were >0.05 (S2 Table). Therefore, we used the consistency-type model for data analysis. After 200,000 simulation iterations, the PSRF value was 1, which indicated that approximate convergence was achieved. Pooled network OR values indicated that the absorbable suture (network OR, 0.37; 95% CrI: 0.19 to 0.70) were superior to staple (Fig 4C). Despite being equivalent to glue suture, the surface score under the cumulative ranking curve analysis (SUCRA) showed that the top-ranked intervention for skin separation were barbed suture (SUCRA score: 58.6, network OR: 0.11, 95% CrI: 0.00–14.35; Fig 4C).

#### NMA for wound complications

**Fig 5:**
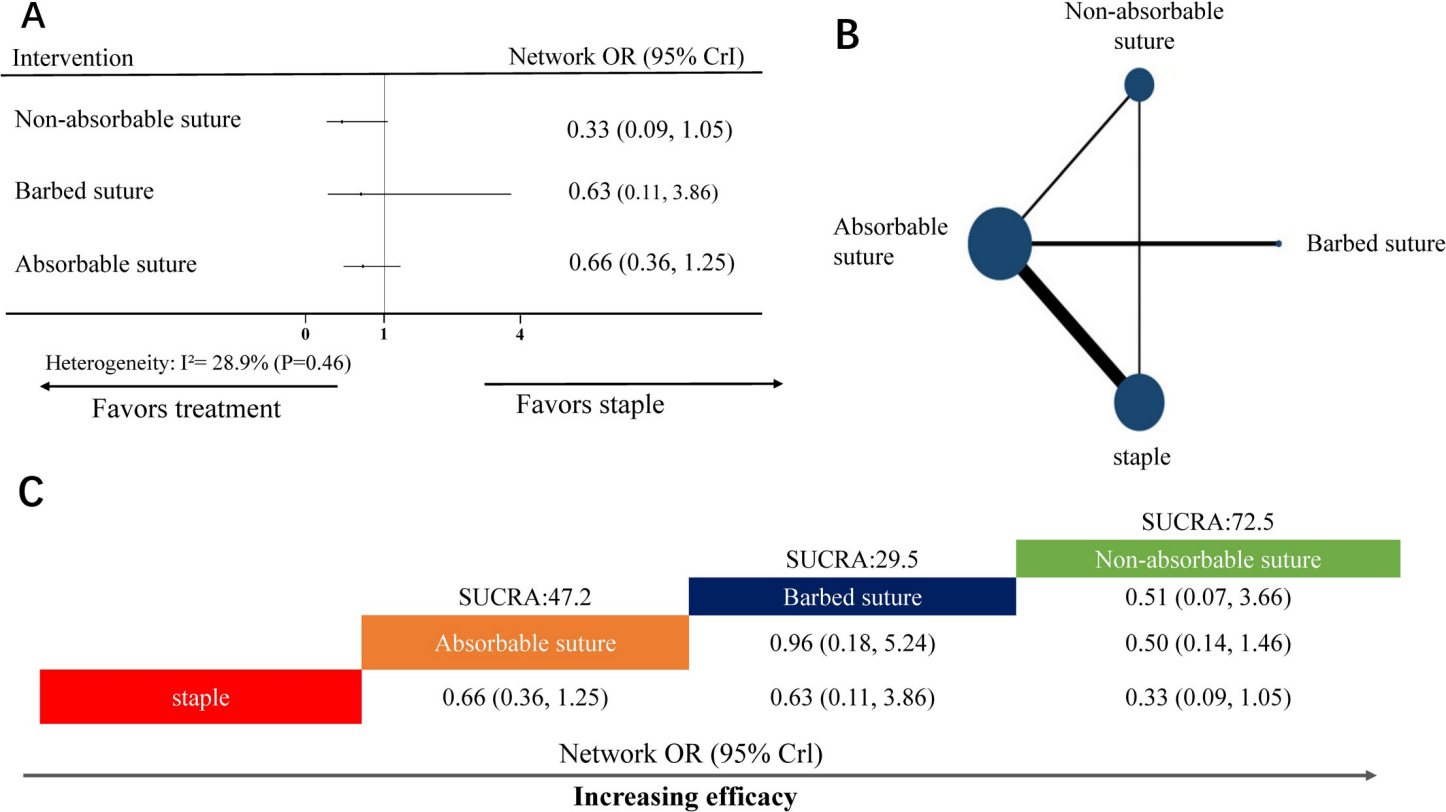
NMA for wound complications. (A) Forest plot of the network meta-analysis comparing each intervention against staple. (B) Each node (blue circles) represents a unique skin closure material; moreover, the size of each node represents the included pregnant woman for the intervention. The connecting line indicates direct comparisons between both nodes. The width of each line represents the number of direct comparisons between interventions. (C) Schematic detailing the most efficacious skin closure materials in NMA for wound complications, and surface under the cumulative ranking curve analysis (SUCRA) score. NMA, network meta-analysis; OR, odds ratio; CrI, confidence interval.

In the NMA of wound complications, 17 RCTs [13,15,17,19,21–23,25–30,34,36–38] (15 two-arm studies, 2 three-arm studies) covering four skin closure materials (Fig 5A) were included. Nine nodes were included in the NMA. Each node represented a unique skin closure material; furthermore, the size of each node represented the included patients for the intervention (Fig 5B). Absorbable suture (19 head-to-head comparisons) and staple (15 head-to-head comparisons) were the most investigated skin closure material.

Heterogeneity analysis indicated no heterogeneity (I^2^-value = 28.9%, *P*-value = 0.46) (Fig 5B). Therefore, we used the random effect model for data analysis.

In the NMA, the node-splitting analysis showed that both *P*-values were >0.05 (S3 Table); therefore, we used a consistency-type model for data analysis. After 50,000 simulation iterations, the PSRF value was 1, indicating approximate convergence. Pooled network OR values showed that no interventions were superior to staple (Fig 5C). Despite being equivalent to staple, the SUCRA score showed that the top-ranked intervention for wound complications were non-absorbable suture (SUCRA score: 72.5, network OR: 0.33, 95% CrI: 0.09–1.05; Fig 5C).

## Discussion

In this network meta-analysis of 26 randomized controlled trials, five different interventions using skin closure materials in more than 8539 women undergoing cesarean delivery were compared. We evaluated the effects of skin closure materials after cesarean delivery on time to skin closure of dermal and epidermal layer, skin separation rate and wound complications. The results of our network meta-analysis show that absorbable suture is still the best choice at this stage. Even the staple can shorten the wound closing time. However, compared with absorbable suture, staple will increase the incidence of skin separation. Compared with ordinary suture, the outcome index of barbed suture was not statistically significant. It is worth noting that all the four studies we included in the barbed suture were absorbable sutures. However, in glue suture, only one trial recruited a small number of patients. The largest number of patients were tested and recruited. Skin closure materials include absorbable suture and staple.

The two most compared skin suture methods during caesarean section are non-absorbable staple and absorbable subcutaneous suture. Dhanya Mackeen et al [39] conducted a systematic review in 2012 reported that there is no conclusive evidence of how the skin should be closed after caesarean delivery. Previous meta-analysis showed that [8,9] compared with staple, absorbable suture significantly reduces the risk of wound complications, but it will be more time-consuming. This is consistent with our research. Barbed suture when suturing tissue, these barbs pierce into the tissue and lock it in place. There is no need to tie the suture. They can reduce the wound closing time and improve the operation efficiency. Recently, Agarwal et al [5] In comparison with the use of barbed suture and absorbable sutures in cesarean delivery, it is shown that the barbed suture can replace the absorbable common suture, which can reduce the time of closure and incidence rate of wound complications. However, these studies only focused on the comparison between barbed suture and ordinary suture. In contrast, NMA combines many published RCTs, which have a broader basis, comprehensively evaluate several types of skin closure materials, and integrate direct and indirect comparisons. And from the cost effect analysis, the cost of barbed suture is much higher than that of ordinary suture [5]. This study has clinical significance because it qualitatively compares the selection of appropriate skin closure materials to close the wound during cesarean delivery and provides a reference for obstetricians.

This study has several limitations. First, like all secondary analyses, NMA should only be combined with the results of similar studies. It is difficult to quantify the factors leading to non-statistical heterogeneity (e.g., study differences in national environment); Therefore, there may be unknown deviations. Secondly, previous meta-analysis [40]. The single-layer and double-layer closure of uterine incision after cesarean delivery was compared with cesarean scar defect and uterine dehiscence and rupture in subsequent pregnancy. There was no significant difference between single-layer and double-layer closure. Therefore, the analysis of this aspect needs to be further studied. We did not conduct subgroup analysis according to the material type of staple and suture. Nevertheless, NMA may produce different results and may require further research.

## Conclusion

In conclusion, our network meta-analysis showed that the risk of skin separation with absorbable suture after cesarean delivery was reduced compared with staple, and does not increase the risk of wound complications, but the wound closure time would slightly prolonged.

## Supporting information

S1 Checklist PRISMA NMA checklist of items to include when reporting a systematic review involving a network meta-analysis. (DOCX)

S1 Table (DOCX)

S2 Table (DOCX)

S3 Table (DOCX)
