# Supplementary material for: Comparison of the effect of skin closure materials on skin closure during cesarean delivery
Source: PLoS One. 2022 Jun 30;17(6):e0270337. doi: 10.1371/journal.pone.0270337 (PMC9246200; doi:10.1371/journal.pone.0270337)
Supplement: S1 Table — (DOCX) [file pone.0270337.s002.docx]

| **Name** | **Direct Effect** | **Indirect Effect** | **Overall** | **P-Value** |
| --- | --- | --- | --- | --- |
| Non-absorbable suture vs Staple | -290.71 (-460.87, -121.88) | -325.57 (-499.16, -163.27) | -313.81 (-437.14, -192.59) | 0.73 |
