# Supplementary material for: Comparison of the effect of skin closure materials on skin closure during cesarean delivery
Source: PLoS One. 2022 Jun 30;17(6):e0270337. doi: 10.1371/journal.pone.0270337 (PMC9246200; doi:10.1371/journal.pone.0270337)
Supplement: S2 Table — (DOCX) [file pone.0270337.s003.docx]

| **Name** | **Direct Effect** | **Indirect Effect** | **Overall** | **P-Value** |
| --- | --- | --- | --- | --- |
| Non-absorbable suture vs Staple | 1.32 (-0.88, 3.59) | 0.91 (-0.59, 2.46) | 1.06 (-0.18, 2.36) | 0.70 |
