# Supplementary material for: Comparison of the effect of skin closure materials on skin closure during cesarean delivery
Source: PLoS One. 2022 Jun 30;17(6):e0270337. doi: 10.1371/journal.pone.0270337 (PMC9246200; doi:10.1371/journal.pone.0270337)
Supplement: S3 Table — (DOCX) [file pone.0270337.s004.docx]

| **Name** | **Direct Effect** | **Indirect Effect** | **Overall** | **P-Value** |
| --- | --- | --- | --- | --- |
| Non-absorbable suture vs Staple | 1.51 (0.05, 3.21) | 0.83 (-0.64, 2.29) | 1.12 (-0.05, 2.39) | 0.42 |
